# Dynamic Geochemistry of Tetraethyl Lead Dust during the 20th Century: Getting the Lead In, Out, and Translational Beyond

**DOI:** 10.3390/ijerph15050860

**Published:** 2018-04-26

**Authors:** Howard W. Mielke

**Affiliations:** Department of Pharmacology, Tulane University School of Medicine, 1430 Tulane Ave-SL-8683, New Orleans, LA 70112, USA; hmielke@tulane.edu; Tel.: +1-504-988-3889

**Keywords:** catalytic converter, children’s lead exposure, geochemical lead imbalance, lead-dust contamination, legacy lead, rapid phasedown, tetraethyl lead (TEL), urban environments

## Abstract

This commentary provides a brief overview of policy decisions that permitted getting tetraethyl lead (TEL) into petrol; global geochemical lead-dust deposition evidence; 1975 catalytic converter requirements; concern about habitability of cities; a personal perspective on legacy lead research that accelerated getting TEL out of petrol; and translational beyond, including New Orleans pre- vs. post-Hurricane Katrina observations about legacy lead interventions that effectively improve urban children’s health outcomes.

## 1. Getting the Lead in

### Early Warnings about Getting Lead Additives into Petrol, 1925

Tetraethyl lead (TEL) was promoted by General Motors and subsidiaries beginning in the early 1920s as an additive to petrol [1]. On 21 April 1925, Yale professor and physiologist Yandell Henderson gave a speech at the American Society of Safety Engineers and International Safety Council. The New York Times covered his speech [2]. Henderson, addressing lead additives to petrol, stated: “…conditions will grow worse so gradually, and the development of lead poisoning will come on so insidiously...that leaded petrol will be in nearly universal use before the public and the government awakens to the situation.” In the same speech, Henderson went on to say: “This is probably the greatest single question in the field of public health that has ever faced the American public. It is the question whether scientific experts are to be consulted, and the action of the government is guided by their advice; or whether, on the contrary, commercial interests are to be allowed to subordinate every other consideration to that of profit”.

The hearing that determined the destiny of TEL as a fuel additive took place on 20 May 1925. The hearing was conducted under the auspices of the U.S. Treasury Department and the U.S. Public Health Service [3]. In his testimony, Henderson raised concerns about medical abilities required to diagnose symptoms of inhaling lead dust compared with ingesting of lead. 

As Henderson stated: “…not one good practicing physician in 1000 would recognize a slight degree of tetraethyl lead poisoning when the lead is inhaled. If he had a good medical education and if he knew the man had a stomach ache and certain other symptoms of lead poisoning, he would size it up as lead poisoning. But if the material is inhaled—and its symptomology is altered when it is inhaled, because of its wider distribution in the body—it is extremely likely that 999 ordinary physicians out of 1000 would fail to recognize the condition as lead poisoning”. Henderson went on to say: “And so I think it is fairly clear that we cannot expect an ordinary practicing physician to recognize the symptoms of tetra ethyl lead poisoning at this time”.

**Figure 1:**
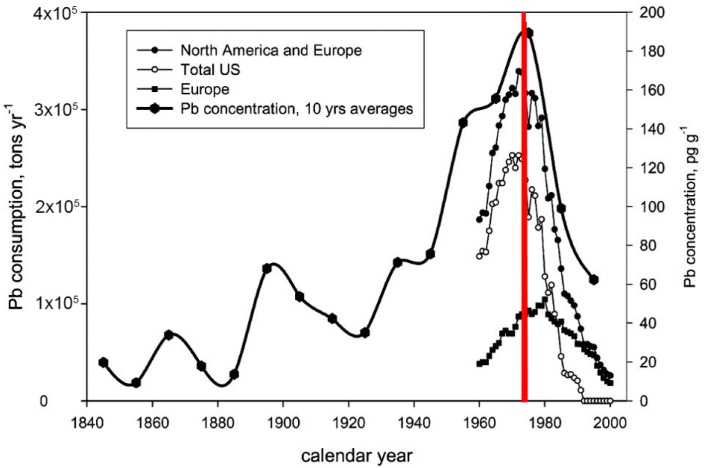
The increase of lead dust accumulation in Arctic snow before and after the U.S. 1975 (red line) catalytic converter installation requirements for all new cars. European countries made the same requirements in 1980. Modified from [4].

Henderson’s (and other scientists’) concerns were not heeded, and TEL became an additive to petrol in 1925. Also, as Henderson predicted in 1925, by 1950 TEL became nearly universal as an additive to petrol. See Figure 1. 

## 2. Getting the Lead out 

### 2.1. Evolving Global Perspective on Lead Contamination, 1965

The scientific capability to evaluate the environmental health effects of TEL lagged far behind the industrial capacity to produce TEL and contaminate the Earth’s air, water, soil, and urban environments. Prior to the 1940s it was impossible to measure lead at small enough quantities to understand TEL’s environmental and health effects. The evolution and application of improved analytical instrumentation for clinical studies drove the reduction of lead exposure guidelines [5]. 

In the early 1940s, the mass spectrometer was created for the Manhattan Project atomic bomb task. Alfred O.C. Nier invented and perfected the mass spectrometer to separate U235 from U238 [6]. The mass spectrometer provided the analytical capability for geochemical study of global Pb contamination. Clair Patterson applied the mass spectrometer and his pioneering cleanroom technology to measure lead in environmental and biological samples [7]. Meanwhile, as shown in Figure 1, the use of TEL rose to ever increasing annual quantities from the 1960s to early 1970s.

### 2.2. The Catalytic Converter, 1975

By the 1970s, carbon monoxide and hydrocarbon releases from automobile exhaust caused an urban air pollution crisis, and it became essential to restrict these exhaust wastes. The catalytic converter was introduced to curtail the emission of these pollutants [8]. Ironically, the reason for removing TEL from gasoline was due to lead ‘poisoning’ destroying the usefulness of catalytic converters. The 1975, requirement for catalytic converter installation on new vehicles fueled with unleaded petrol, resulted in a rapid decrease of lead in the high arctic snow samples. See Figure 1.

### 2.3. Urban Geochemistry and Health, 1980

Clair Patterson contributed a chapter to the National Academy of Science report entitled “Lead in the Human Environment” [9]. Patterson made an ominous prediction about the human health effects of lead in urban environments when he wrote: 

“Sometime in the near future it probably will be shown that the older urban areas of the United States have been rendered more or less uninhabitable by the millions of tons of poisonous industrial lead residues that have accumulated in cities during the last century” [9].

Were Patterson’s inhabitability concerns warranted? Patterson did not present empirical data about the amount of lead in urban environments or the connection between lead contamination of cities and health effects of urban lead dust accumulation. In 1979, Herbert Needleman [10] presented evidence on health damage by demonstrating that varying amounts of lead accumulation in school children’s deciduous teeth were associated with decreased learning abilities in the classroom. An evidence-based link between urban lead dust, habitability, and health was required to evaluate Patterson’ prediction. 

### 2.4. Baltimore Urban Garden Project, 1983

The Baltimore Urban Garden (BUG) project was pursued, in part, because of a 1974 Environmental Health Perspectives article published by personnel employed by the Ethyl Corporation. Ter Haar and Aronow concluded: 

“…it is clear that nearly all of the lead in dirt around these houses is due to paint from the houses. Lead antiknock additives are therefore not a significant contributor to the lead content of dirt around houses where children usually play” [11]. 

My personal involvement with the lead issue began in 1976 with the development and management of the BUG study. By the mid-1970s, several technical advances made it possible for investigators to conduct urban geochemistry research. First, an affordable and reliable atomic absorption spectrometer (AAS) became available for analytical quantification. Second a data-dependent statistical model to evaluate skewed, spatial data collected from metropolitan and rural Baltimore. Also, computer hardware and software evolved to calculate permutations on relatively large datasets.

The BUG project was conducted in collaboration with Rufus Chaney at the U.S. Department of Agriculture (USDA). The first task focused on developing a safe and effective method for soil metal analysis. USDA extraction methods using hydrochloric acid were initially employed. Numerous trials on soil samples from the city indicated a need for adjustment of the method. For example, the longer the shaking time the lower the quantity of lead. Dr. Chaney recognized that lead solubility of hydrochloric acid (HCl) was limited; as a result, 1 molar nitric acid (NO_3)_ was substituted for HCl to increase Pb solubility; the ratio of soil to acid was 1:5 (later changed to 1:50 with a more sensitive analytical instrument); and a shaker time of 2 h was selected for room temperature extraction. It took over a year to satisfactorily complete the analysis of the BUG soil samples for lead, four other metals (Cd, Cu, Ni, Zn), and soil pH. 

An essential rule for Analysis of Variance (ANOVA) is the requirement for a normal distribution. A USDA statistician worked on the data for a few days and returned the results. The highest “outliers” were “trimmed” and other manipulations were made so that the data fit the “normal curve” requirement of the ANOVA statistical model [12]. Because our interest was in the actual data that we worked long hours to obtain, we sought an alternative statistical model that was data dependent, not subject to the requirements of the model. 

Serendipity played a major role in the analysis of the BUG results. Statistician Paul Mielke, Jr. (my brother) and Ken Berry are pioneers in adapting R.A. Fisher’s original permutation models into software for modern computers [13]. Multi-response permutation procedure (MRPP) was used to evaluate the geographic differences between the high lead soils and the low lead soils of Baltimore [14]. The results showed that the probability for chance alone for the observed differences was extremely small (*p*-value = 10^−23^) between high lead in the interior of the city compared with low lead in outlying areas of the city. Based on the Baltimore results, we predicted that all larger cities of the U.S. would have a similar pattern of lead distribution [14]. The results provided support for Patterson’s prediction. 

The major remaining question was whether the amount of lead contamination of community soils influenced children’s exposure. Clinical data on children’s lead exposure was not available for the BUG study. A more extensive project was needed to evaluate the association between soil lead and blood lead. Minnesota was the next study location.

### 2.5. Minnesota Legislature Children’s Blood Lead and Soil Lead Project, 1986–87

The association between children’s exposure and lead contamination of soil was undertaken through the efforts of a coalition of citizens and the Minnesota Legislature. The legislature funded the Minnesota Department of Health to conduct a blood lead screening survey and the Minnesota Pollution Control Agency (MPCA) to survey soil lead. Both projects were conducted within the same residential communities (census tracts) throughout the state. The soil lead results alone were reported by employees of the MPCA [15]. The associations between children’s lead exposure and soil lead, accounting for community characteristics, including city size, location, traffic flows, and home age were reported independently [16,17]. 

The results strongly supported Patterson’s concern about the inhabitability of larger cities. It also demonstrated a strong association between the amount of lead accumulated in the soil and children’s blood lead within the same communities. Based on the results, the following statement summarized the situation: 

“Improvements in the quality of urban residential environments are necessary in order to broaden the margin of safety to prevent lead toxicity for the most exposed populations of children in Minnesota” ([16] p. 268).

The studies in Minnesota supplied evidence-based science which showed that accumulated vehicle lead dust from TEL in petrol was a major source of children’s lead exposure from the air and soil environment. The Minnesota Legislature sought to ban the use of TEL in petrol. However, according to federal laws that action was not legal. The response of the Minnesota Legislature was to petition Congress to ban TEL in petrol. The hearing took place on 22 June 1984. On behalf of the citizens of Minnesota, I was invited to make the case at the hearing to ban TEL (Supplement 1). Patrick Reagan wrote the technical report to the EPA that supported the TEL ban. The EPA response was to establish the rapid phasedown regulations on TEL in petrol for highway use on 1 January 1986. 

The results of children’s blood lead before and after the 1975 catalytic converter requirement and the 1 January 1986 rapid phasedown of TEL demonstrated a remarkable decrease in blood lead [18]. This result was further evidence that lead dust derived from TEL additives were a major driver of children’s lead exposure. The ominous predictions about the negative effects of TEL on health by Henderson in 1925 and Patterson in 1980 weredocumented. The critical question at this time concerns actions that translate scientific understanding into improving urban environmental health.

## 3. Translational Beyond

### Intervention Lessons from Hurricane Katrina, New Orleans, 2005

In 1990, soil lead and children’s exposure studies were instituted at Xavier University of Louisiana, New Orleans. The studies were well-funded by the U.S. Agency for Toxic Substances and Disease Registry. Prior to the ravaging flood by Hurricane Katrina in 2005, the entire metropolitan area was mapped [19]. Ten years after the flood, the city soils were mapped again [20,21]. In addition to mapping the soil lead, children’s (≤6 years old) blood lead data was collected before and after Hurricane Katrina by the Louisiana Health Housing and Lead Poisoning Prevention program. 

**Figure 2:**
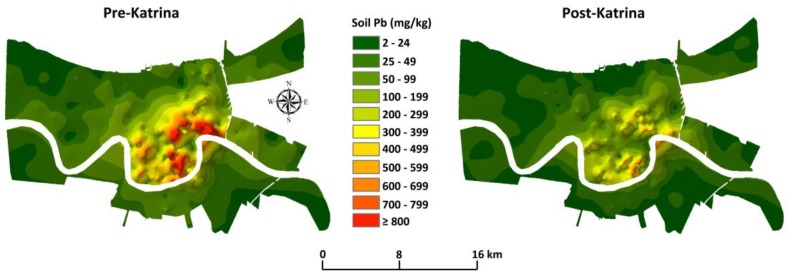
Pre- and post-Hurricane soil lead map by medians for 285 census tracts of New Orleans. Note the legend showing the shift from dark red, higher soil lead in pre-Katrina to orange and yellow colors representing substantially lower soil lead in post-Katrina New Orleans.

Figure 2 shows before and after maps of the Hurricane Katrina dynamic soil lead changes in metropolitan New Orleans. Hurricane Katrina flooded about eighty percent of New Orleans. Decreases in soil lead can be attributed to the Hurricane Katrina storm surge. The storm surge transported massive amounts of low-lead coastal sediment into New Orleans. 

**Figure 3:**
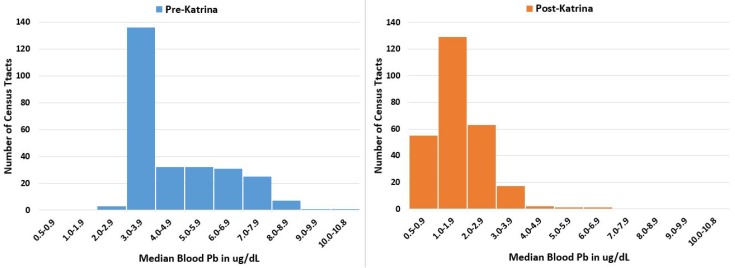
Histograms of children’s (≤6 years old) median blood lead levels before and after Hurricane Katrina. *N* = 268. Not all census tracts had sufficient numbers of tested children for matching between the two surveys.

Laidlaw and Filipelli [22] presented a paradigm of the soil reservoir and seasonal resuspension as an ongoing inhalation lead exposure source both outside and inside homes. According to their model, lead burdens to children are based on the reservoir of soil lead combined with atmospheric parameters that drive particulate resuspension. As a result, there is a strong seasonal relationship between atmospheric particulate loading and blood lead levels in children. While seasonality of exposure is not included in New Orleans, Figure 3 illustrates the integrated blood lead response of children in metropolitan New Orleans in response to changes in soil lead (illustrated in Figure 2). The histograms of children’s blood lead data are matched and stratified by communities (census tracts) for pre-and post-Hurricane Katrina. The post-Katrina results show substantial progress towards improving the environment for children. This supports the Laidlaw and Filipelli [22] paradigm and indicates that soil lead is a driver of children’s lead exposure. Note that the peak soil in Figure 2 and highest blood lead in Figure 3 indicate that children living in some communities continue to experience excessive lead exposure. 

**Figure 4:**
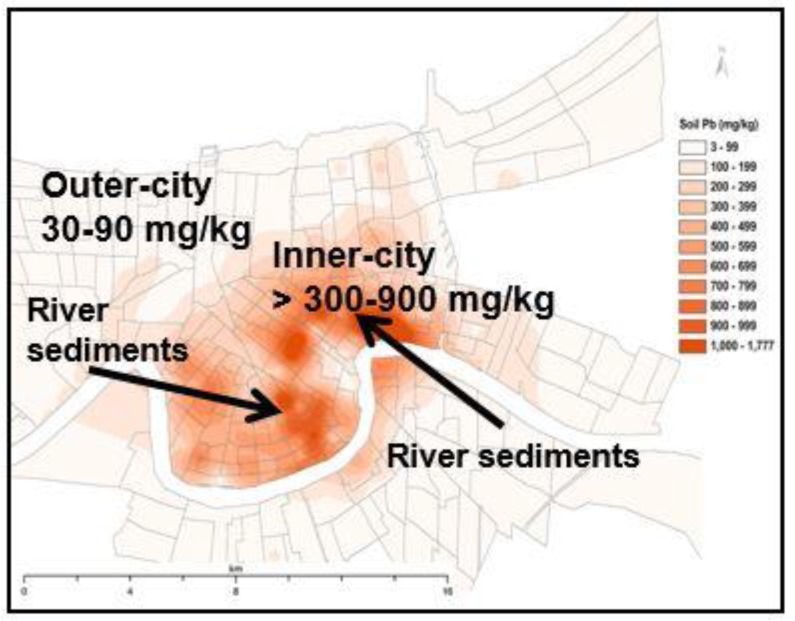
The dynamic changes experienced before and after Hurricane Katrina indicate that a surface covering of low lead (~5 mg/kg) alluvial sediment on lead contaminated communities is translational as a feasible and cost-effective method for reducing children’s lead exposure.

Outlying areas of New Orleans, shown in Figure 2, contain low lead soils. This is a common pattern for all cities. The experience from Hurricane Katrina reveals methods that communities can take to address legacy lead and reduce lead exposure of children. As illustrated in Figure 4, translational efforts to transport low lead soil into lead contaminated communities can improve childcare centers, parks, and other properties where children play [23,24]. The clean soil proposal is not new. Norway established a national precedence for primary prevention actions to clean up soil contaminants [25].

## 4. Conclusions 

The global geochemical experience with TEL is a precautionary narrative about humanity’s capacity to unwittingly contaminate the Earth’s air, water, and soil. Many urban soil lead studies indicate that Clair Patterson’s dire prediction about the inhabitability of larger cities was warranted. Urban environments where most of humanity resides require special consideration. Early childhood lead exposure has multiple adverse health consequences including cognitive and behavioral discrepancies between citizens living in various communities. Hurricane Katrina flooded 80% of New Orleans and demonstrated restorative actions that reduce children’s lead exposure and bolster wellness of citizens living in urban environments. All cities have low-lead soils within their outlying areas and children would benefit from low lead soil landscaping informed by urban soil lead surveys. The translational prospects of landscaping residential areas, childcare centers, parks, and elementary school properties is possible but requires public will. 

## Supplementary Materials

The following are available online at http://www.mdpi.com/1660-4601/15/5/860/s1, Figure S1: 1984 Senate Hearing Mielke testimony.
