# Supplementary material for: Dynamic Geochemistry of Tetraethyl Lead Dust during the 20th Century: Getting the Lead In, Out, and Translational Beyond
Source: Int J Environ Res Public Health. 2018 Apr 26;15(5):860. doi: 10.3390/ijerph15050860 (PMC5981899; doi:10.3390/ijerph15050860)

Printed for the use of the Committee on Environment and Public Works

Airborne Lead Reduction Act of 1984.
Hearing before the Committee on
Environment and Public Works.
United States Senate, Ninety-Eighth
Congress, Second Session on S.2609.
A Bill to Amend the Clean Air Act With
Regard to Mobile Source Emission Control.
June 22, 1984.

Pages 44-46.

STATEMENT OF DR. HOWARD W. MIELKE, DEPARTMENT OF GEOGRAPHY, MACALESTER COLLEGE, AND MEMBER OF THE LEAD COALITION OF MINNESOTA

Dr. MIELKE. Thank you very much, Mr. Chairman. I am Howard Mielke. I am a teacher, a researcher, a parent, an urban citizen. I represent the Lead Coalition. I appreciate the opportunity to appear before this committee to discuss our understanding of the seriousness of the lead problem.

The Lead Coalition is an organization that represents a number of civic and environmental groups and individual citizens in Minnesota. The group formed when it became clear that the need for a low-lead environment for our children was not being met. We are parents and adults who speak for the children in our society who cannot speak for themselves.

I will summarize my statement by making three points concerning lead in the city, urban lead and children, and the urgency for acting immediately to ban the major aerosol source of lead: leaded gasoline.

My first point is that the current combination of public highways and the use of leaded gasoline causes an accumulation of lead in the heart of our major cities. I have with me some soil samples from play areas in Minneapolis in an inner-city community called Cedar Riverside. But these soils could be from Manchester, VT; Boise, ID; Seattle, WA; Dallas, TX; or Fargo, ND—every major city in the United States.

The samples came from a densely populated area, an urban community that has very high traffic flows through it. And in the appendix to my statement I compare these samples from Cedar Riverside with some samples that were collected around a community with a lead-producing plant, also in Minneapolis.

The State of Minnesota cited the lead plant because it was contributing too high an air emission to the environment. Yet when you look at the ambient environmental lead levels, you find that the inner city has twice the levels as were found in the neighborhood of the lead plant.

Why has this happened? In Minnesota we have around 10,000 pounds of lead aerosols from secondary smelters and producers per year which is being controlled by the State.

By comparison, motor vehicles emit 2 million pounds of lead aerosols in Minnesota per year. These aerosols are subject to Federal laws and the State and municipalities are prohibited from doing anything about them.

Minneapolis receives about 300,000 pounds of lead aerosols per year from vehicle exhaust. The amount of lead aerosols in Minnesota—and in fact, 28 other States—increased in 1983 as a result of changes in Federal standards. The increase in Minnesota was 15 to 20 percent.

So who is being protected by the present law and the present standards? In Minnesota, we can do something about lead aerosols emitted from secondary smelters, which are very small, but we can't do anything about the enormous emissions from the major source, lead aerosols from gasoline.

My second point concerns 300,000 pounds of lead aerosol dust per year affect the people of the city.

Senator STAFFORD. You have about 1 minute remaining.

Mr. MIELKE. Not all people are equally sensitive. Children are the major victims. And it turns out that very small amounts tenths of grams—of this soil from play areas of Minneapolis can potentially create serious health problems to children who regularly play in these soils and suck their thumbs.

All children are vulnerable to exposure, and one of the situations we find, for example, is that children love parades. On June 2, my family and I went to a parade along Grand Avenue, which is a very heavily traveled avenue. The children sit on the curbside, and watch the floats go by. Candy and bubblegum one thrown from the floats. And thus we find children with very sticky fingers sitting on the curb with their hands constantly going back and forth, between their mouths and the ground. The soil lead levels of Grand Avenue are on the order of 500 to 1,000 parts per million.

So what we start out with as fun and frolic, ends up being a tragic situation where children are being exposed to lead aerosols that have accumulated in the city.

My third point is that because children are most sensitive to lead during the first years of life, waiting 4 more years to ban leaded gasoline will expose another generation of children to execessive lead levels. Since the learning ability of thousands of children and future citizens are being decreased by lead, there is some urgency to acting sooner.

Senator STAFFORD. Could you summarize quickly?

Mr. MIELKE. Yes. In summary, I want to say that the city has a serious design flaw that causes lead to accumulate in it; that the major source of new lead aerosols are leaded gasoline; that Federal law prohibits States and cities from controlling these lead sources; that children, urban children are at the greatest risk; that as a result of the excessive exposure to lead, children are being robbed of a major resource, their health and their mental abilities; that there seems to be no engineering or economic justification to keep lead in gasoline.

The needs of the children of our society should take precedence in order to have a bright future. And with that, I would close my statement.

Senator STAFFORD. Thank you. Thank you very much, Dr. Mielke.

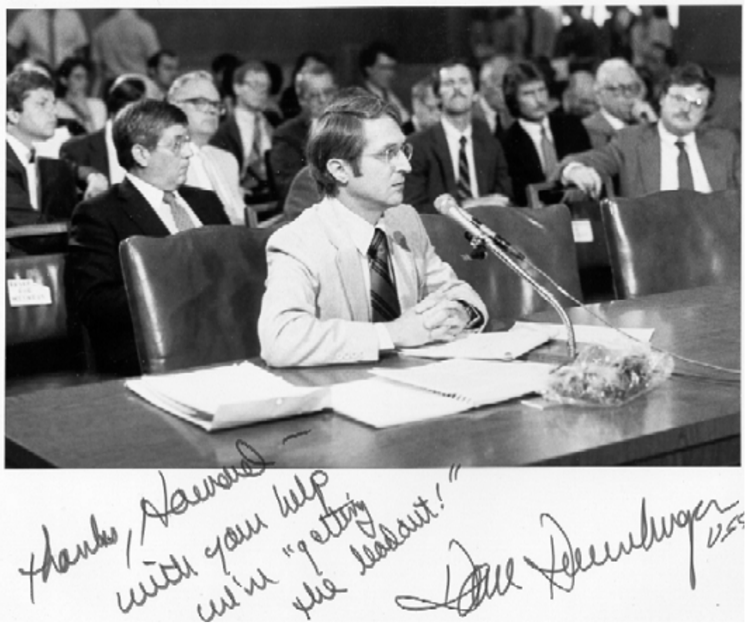

Supplement: Supplementary file 1 [file ijerph-15-00860-s001.pdf]
